# Prenatal SSRI Exposure Increases the Risk of Autism in Rodents via Aggravated Oxidative Stress and Neurochemical Changes in the Brain

**DOI:** 10.3390/metabo13020310

**Published:** 2023-02-20

**Authors:** Ramesa Shafi Bhat, Mona Alonazi, Sooad Al-Daihan, Afaf El-Ansary

**Affiliations:** 1 Biochemistry Department, College of Science, King Saud University, Riyadh 11495, Saudi Arabia; 2 Central Research Laboratory, Female Campus, King Saud University, Riyadh 11495, Saudi Arabia

**Keywords:** SSRI, autism, oxidative stress, neurotransmitters, fluoxetine

## Abstract

The mechanisms underlying selective serotonin reuptake inhibitor (SSRI) use during pregnancy as a major autism risk factor are unclear. Here, brain neurochemical changes following fluoxetine exposure and in an autism model were compared to determine the effects on autism risk. The study was performed on neonatal male western albino rats which were divided into Groups one (control), two (propionic acid [PPA]-induced autism model), and three (prenatal SSRI-exposed newborn rats whose mothers were exposed to 5 mg/kg of fluoxetine over gestation days 10–20). SSRI (fluoxetine) induced significant neurochemical abnormalities in the rat brain by increasing lipid peroxide (MDA), Interferon-gamma (IFN-γ), and caspase-3 levels and by depleting Glutathione (GSH), Glutathione S-transferases (GST), Catalase, potassium (K+), and Creatine kinase (CK) levels, similarly to what has been discovered in the PPA model of autism when compared with control. Prenatal fluoxetine exposure plays a significant role in asset brain damage in newborns; further investigation of fluoxetine as an autism risk factor is thus warranted.

## 1. Introduction

Serotonin (5-hydroxytryptamine; 5-HT) is a monoamine neurotransmitter that controls various neuronal interactions through specific transporters, receptors, and intracellular signaling pathways [1]. It is one of the first neurotransmitters to emerge in the mammalian brain. Serotonergic neurons start at five weeks old in human embryo and proliferate until ten weeks of gestation, increase for the first 2 years after birth, and decrease to reach adult levels at five years of age. Serotonergic activity can shape the develop neuronal circuitry and is involved in plasticity during the perinatal period [2]. Serotonin signaling pathways are important for normal brain functions in sensory processing, regulating emotion and motor activity in addition to being involved in morphogenetic activities during brain development including neural cell proliferation, migration, and differentiation. It reduces neural plasticity and acts as a nervous tissue growth factor in the developing brain [3]. It is also involved in several developmental processes, such as cell division, neural migration, cell differentiation, synaptogenesis, and early cortex development [4]. Reduction in central serotonin levels can result in various psychiatric disorders such as depression, anxiety, aggression, childhood hyperactivity, and some behavioral disorders [5]. Thus, most of the antidepressants prescribed these days are among the selective serotonin reuptake inhibitors (SSRIs), which can increase the levels of extracellular serotonin in the brain by blocking its reabsorption. Some members of the SSRIs group of drugs not only decrease the uptake of serotonin but are reported to inhibit the uptake of some other neurotransmitters as well. Sertraline inhibits dopamine reuptake; fluoxetine inhibits noradrenaline and dopamine; imipramine inhibits both noradrenaline and serotonin reuptake [6]. Although SSRIs are repeatedly used during pregnancy, they can be teratogenic and increase the risk of premature delivery, low birth weight, neonatal cardiovascular abnormalities, and offspring metabolic disorders [7]. Impaired serotonin-dependent shaping of neural circuitry in SSRIs-exposed babies might increase the risk of neurodevelopmental disorders. Magnetic resonance imaging has reported hyper-connectivity in auditory resting-state networks in infants exposed prenatally to SSRIs in comparison to healthy control infants [8].

Many studies have reported the associations of prenatal depression with the brain development of infants. Depression during pregnancy is a major health issue not only for mothers but also for newborns. Children of depressed females during pregnancy are four times more likely to be diagnosed with depression in their teenage years than those unexposed to antenatal depression. Maternal anxiety in pregnancy is associated with lower mental development scores, hyperactivity, and behavioral and emotional problems in children. Most women use antidepressants during pregnancy as they experience depression and anxiety disorders in their reproductive years [9]. SSRIs are among the most prescribed medications for maternal depression as it mainly targets the serotoninergic system [10]. SSRIs can pass the human placenta to reach the fetus, and they can also be consumed by neonates through nursing because their active metabolites are excreted into human milk [11]. Many studies have reported neurological issues and other health problems in children exposed to SSRIs before birth. Recently, Zengeler et al. [12] reported a strong inflammatory reaction at the maternal–fetal interface due to SSRI treatment during pregnancy. Babies that are exposed to inflammation develop behavioral changes such as diminished communication and decreased interest in social interactions. An immune challenge during pregnancy can lead to permanent brain changes [12]. Many epidemiological reports have linked maternal immune activation with neurodevelopmental disorders, psychiatric conditions, and other neurologic disorders in children [13,14]. Low immunity promotes infections and autoimmune conditions and elevates the chance of mental conditions in offspring [15]. Some animal-based studies reported that exposure to SSRIs during pregnancy can interrupt brain development resulting in neurodevelopmental abnormalities [16,17]. Altered pain response and subtle delays in motor development in SSRI-exposed infants have been reported by Oberlander [18]. Moreover, Croen et al. [19] showed that prenatal SSRI exposure can double the risk of autism in newborns.

Several studies based on neuroimaging and genetics have linked serotonin to the pathophysiology of autism. Autism patients usually have disturbed serotonin levels and altered serotonin end-metabolites such as 5-hydroxy indole acetic acid in their body and cerebrospinal fluid (CSF), respectively [20,21]. Moreover, nearly one third of autistic children have hyperserotonemia [22]. Autism is a neurodevelopmental ailment, characterized mainly by impaired social interaction and repetitive behaviors [23]. The incidence of autism in low to middle-income countries is not defined but internationally, a single child in 160 children is suffering from this disease [24]. Recently, in the US, the Centers for Disease Control and Prevention (CDC) and Autism and Developmental Disabilities Monitoring (ADDM) Network reported that 1 in 44 children is autistic [25]. Autism is more prevalent in boys with co-occurring disorders with symptoms including anxiety, epilepsy, depression, disturbed sleep, and self-harm [26]. A lot of research on the etiological factors of autism acknowledged both genetic and environmental factors responsible for the pathogenesis of this disease [27]. Many previous studies have linked genetic factors related to depression and the use of SSRIs with the risk of ASD [28]. SSRI exposure to fetal through maternal blood can affect the developing brain by damaging serotonin receptors through negative feedback known as the developmental hyperserotonemia model of autism [29]. This may be the reason for high levels of serotonin in blood with the low binding potential of the same in various brain sites in autistic patients [30]. Hyperserotonemia is reported as the first biomarker identified in autism and is present in more than 25% of autistic children. Positron emission tomography-based studies for normal healthy children showed elevated serotonin synthesis between two and six years of age followed by a decline at puberty but not in autistic children.

Propionic acid (PPA), a by-product of enteric fatty acid bacterial fermentation, can exert profound effects on the stomach, brain, and behavior. The brain tissue of PPA-treated rats exhibits a wide range of neurochemical abnormalities, including neuroinflammation, glutamate excitotoxicity, oxidative stress, GSH depletion, and altered membrane phospholipids, all of which are present in ASD patients [31,32]. Orally administered PPA (250 mg/kg/body wt.) for 3 days in the rodent model of ASD was repeatedly used by our research team as a valid model that offers the same pathophysiology and the symptoms of ASD behavior [32,33]. All of these findings lead us to consider fluoxetine as an autism risk factor. The neurotoxicity of newborn rats exposed to fluoxetine during pregnancy was compared using a rodent model of autism generated by propionic acid as a vehicle.

## 2. Materials and Methods

### 2.1. Animals

The experiment was started with 10 female western albino rats (180 to 200 g) which were obtained from the animal house of the Pharmacy College of King Saud University. Healthy female western albino rats were mated overnight with normal male rats in the proportion of 1:1, and the day spermatozoa were detected in the vaginal smear was designated the first day of gestation. Each pregnant female rat was individually kept in a cage. Pregnant female rats were randomly divided into two groups. Set I was grown in a normal condition and Set II was treated with 5 mg/kg/body wt. of fluoxetine from pregnancy day 10 to day 20 [34,35]. Male neonatal rats from both sets were differentiated by observing the urinary papilla and genital opening on day four of birth. Twenty male neonatal rats that were born from Set I were further divided into two groups (with ten neonatal rats in each group). Group one received phosphate-buffered saline throughout the experiment and served as the control group; Group two was treated with oral neurotoxic doses of PA (250 mg/kg/body wt.) for 3 days to induce the autistic features [22]. Ten newborn male rats from Set II were assigned to Group three. As a result, the three groups—each consisting of 10 neonatal rats—were set up as follows:

Group one (control): Male pups from Set I were administered an oral dose of 1 mL of normal saline daily.

Group two (positive control/PA rodent model): Male pups from Set I were administered oral neurotoxic doses of PA (250 mg/kg/body wt.) for 3 days at 19 days after birth.

Group three (fluoxetine-treated mothers): Male pups from Set II were administered an oral dose of 1 mL of normal saline daily. On the 22nd day after birth, all groups were killed by decapitation.

Animals were raised under standard conditions at a temperature of 23 °C, humidity of 37%, with light for 12 h. All animal work was conducted in the animal house in the zoology department at the College of Science at King Saud University.

**Figure 1:**
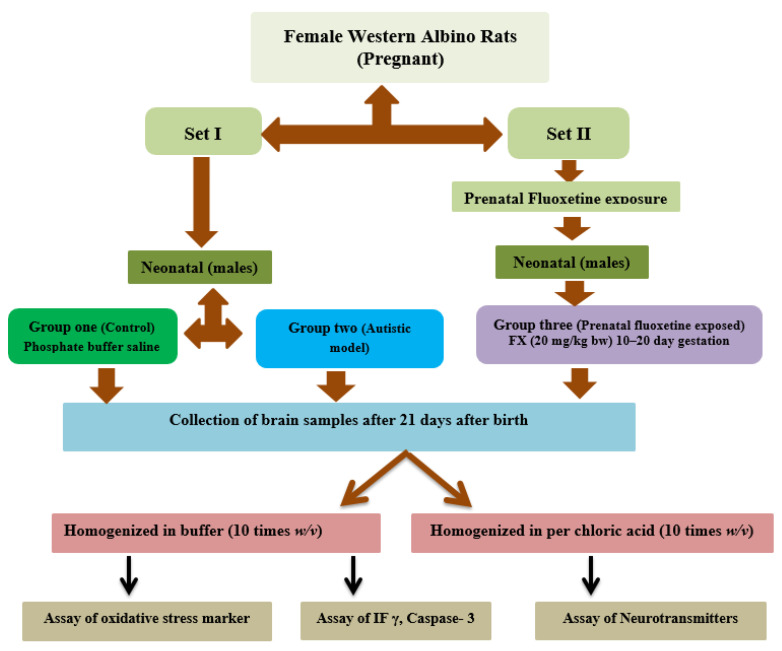
Schematic representation of the different groups with treatment involved in the study.

A schematic presentation of the designed groups is summarized in Figure 1.

The protocols used in the present study were approved by the Ethics Committee at King Saud University (Approval Reference no SE-19-97).

### 2.2. Brain Tissue Collection

The whole brain was collected and washed with cold normal saline and then homogenized in 1:10 weight/volume in double distilled water followed by centrifugation at 3500 rpm for 15 min. The supernatant was collected and used for various biochemical analyses.

### 2.3. Biochemical Analyses

The biochemicals mentioned below were measured in the brain homogenate.

#### 2.3.1. Lipid Peroxidation

The method described by Ruiz-Larrea et al. [36] was used to measure lipid oxidation based on the formation of thiobarbituric acid reactive substances (TBARS), namely malondialdehyde (MDA). Brain tissue samples were boiled with TBA to form a pink chromogen; the absorbance was measured at 532 nm and the concentration was calculated as μmoles/mL using the extinction coefficient of MDA.

#### 2.3.2. Glutathione

The method of Beutler et al. [37] was used to measure glutathione using 5,5′-dithiobis (2-nitrobenzoic acid) and sulfhydryl compounds to produce a relatively stable yellow color that was red at 412 nm. A standard glutathione solution was used for calculating the concentration of glutathione.

#### 2.3.3. Glutathione-S-Transferase

An assay kit from BioVision, USA, using glutathione S-transferase (GST)-catalyzed reaction between glutathione and 1-chloro-2,4-dinitrobenzene was used to measure GST activity which was expressed as nmol/mL [38].

#### 2.3.4. Catalase

Catalase activity was measured using the method described by Chance [39] by monitoring the rate of dissociation of hydrogen peroxide per minute at 240 nm.

#### 2.3.5. Potassium

Potassium levels were measured by producing a colloidal suspension by reaction with sodium tetraphenyl boron which was red at 620 nm [40].

#### 2.3.6. Neurotransmitter

The concentrations of noradrenaline, dopamine, and serotonin were determined in brain homogenates using high-performance liquid chromatography with electrochemical detection [41]. Briefly, Brain tissue was homogenized with 0.1 M perchloric acid and then centrifuged at 10,000× *g* at 4 °C for 30 min. The supernatant was injected into the HPLC instrument with an analytical column of ODS 2 C18, 4.6 × 250 mm protected by a guard column with a particle size of 5 μm. The electrochemical detector model HP 1049 A was used to detect three neurotransmitters and expressed as ng/100 mg.

#### 2.3.7. Creatine Kinase

Creatine kinase was determined using the CU NSC kit from NSC Human, Germany, and the activity is expressed in U/L at 340 nm.

#### 2.3.8. Caspase-3

Caspase-3 was measured using an enzyme-linked immunosorbent assay (ELISA) kit (Cusabio, Wuhan, China) with a detection limit of 0.312 ng/mL–20 ng/mL.

#### 2.3.9. Interferon Gamma

Interferon Gamma was measured using an ELISA kit (Thermo Fisher Scientific, Rockford, IL, USA) with a detection limit of 25.6–1000 pg/mL.

### 2.4. Statistical Analysis

Results were presented as mean ± standard deviation using SPSS and evaluated using one-way analysis of variance test between all groups and with Dunnett’s test. P values of ≤0.05 were considered significant. Receiver operating characteristic (ROC) analysis was performed to measure the effectiveness of the studied parameters in terms of either the neurotoxicity of PA and fluoxetine.

## 3. Results

**Figure 2:**
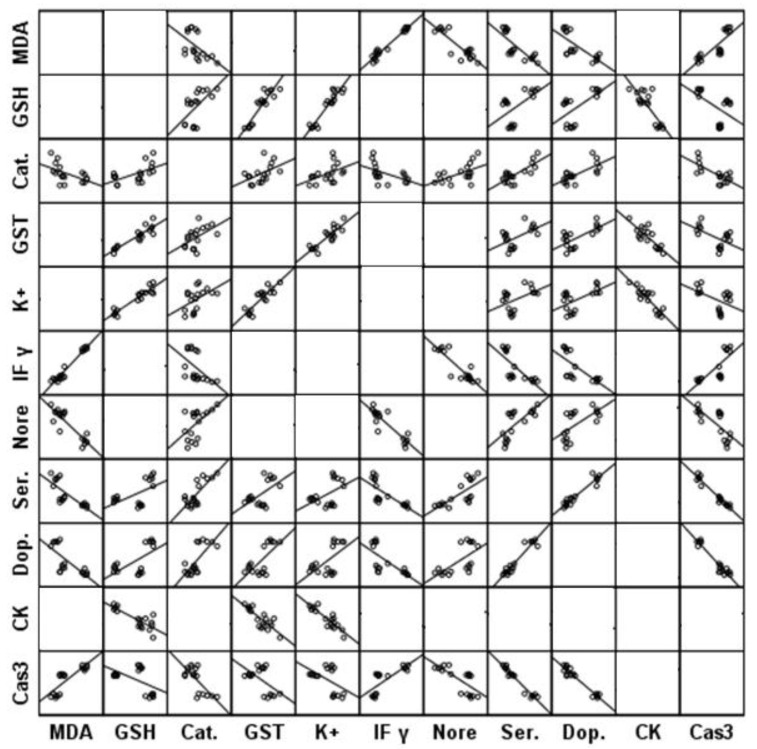
Matrix correlations between all parameters which have significant correlation at 0.05 level.

Table 1 presents the levels and percentage changes relative to control for all the measured parameters in brain homogenates of all three groups under study. Table 1 clearly shows that both PPA and fluoxetine induce a significant increase (*p* ≤ 0.001) in lipid peroxide, IFN-γ, and caspase-3 in rat pup brains compared with the control group. Glutathione, GST, Catalase, K+, and CK were significantly (*p* ≤ 0.001) decreased in both PPA and fluoxetine groups compared with the control group. In addition, all measured neurotransmitters, including noradrenaline, dopamine, and serotonin, were significantly (*p* ≤ 0.001) decreased in groups treated with PA and fluoxetine. Figure 2 represents the matrix correlations between all parameters which have a significant correlation at 0.05 level. Table 2 demonstrates the multiple regression using a stepwise method for all the measured parameters as a dependent variable. Table 3 presents the results of ROC analysis with the area under the curve, specificity, and sensitivity of all measured parameters.

## 4. Discussion

Autism is diagnosed at the age of 3–4 years and persists throughout the life of a patient with symptoms such as shortfalls of social interaction and communication, negligible eye contact, expression of emotions, etc. To date, no effective therapy to treat or prevent autism is available, hence identification of environmental risk factors is extremely needed as a public health goal. The environment may possibly act as one of the major risk factors for various neurodevelopmental disorders including autism. Prenatal SSRI exposure has been linked to an increased risk of autism in babies in a number of recent population-based studies; however, the underlying processes are not fully understood [42,43,44,45]. Due to the lack of human brain tissue samples, animal modeling is the most effective method for understanding the neurochemical alterations in individuals with brain diseases [46]. Thus, we relate the neurochemical changes in the brain caused by exposure to fluoxetine with the PPA-induced autism model to determine whether fluoxetine is a risk factor for autism (Table 1). Our findings revealed that prenatal exposure to fluoxetine could result in the deterioration of brain neurochemistry in almost the same trend as observed in the PPA model of autism.

Concerns about the dangers of prenatal exposure to environmental toxins have resulted in a mother bias in epidemiological studies examining trends in ASD onset [47,48]. Many risk factors related to the development of autism in children are directly linked to oxidative stress [49]. Many studies have linked autism with high oxidative stress mainly due to abnormal metabolism and accumulation of toxins resulting in reactive oxygen species (ROS). Autistic patients have decreased antioxidant capacity, elevated lipid peroxidation, and reduced glutathione in both plasma and primary immune cells. The current results discovered a significant increase in MDA and CK; in addition to a significant decrease in GSH, GST, catalase, and K+ levels in the brain tissue of neonatal pups after intrauterine exposure to fluoxetine, which can be connected with the development of oxidative stress related to autism, many studies have shown that increase in oxidative stress due to maternal exposure to environmental toxins or some drugs during pregnancy have been associated with the development of autism [48,50].

Significant decrease in noradrenaline, dopamine, and serotonin levels was observed in the brain tissues of prenatal fluoxetine-treated rat pups. These neurotransmitters play a major role in the normal brain development and regulate memory, motor activities, and behaviour [51]. Prenatal exposure to fluoxetine has affected the brain neurotransmitter levels of neonates as this drug can cross the placenta and can reach the embryos during the developmental stage. We also observed that the brain tissues of PA-induced rodent model of autism showed abnormal neurotransmitter levels. Our results are in good agreement with multiple studies which reported that drugs provided to pregnant women could disrupt neurotransmitter systems, increasing the likelihood of having a child with autism [43,45,52].

IFN-γ is a T-helper type 1 cytokine, which is expressed in neurons during cell-mediated immune responses, whereas caspase-3 is determined to be essential for normal brain development and is also involved in the programmed cell death of neurons. A significant increase in the IFN-γ and caspase-3 levels was observed in the brain tissues of rat pups exposed to fluoxetine during pregnancy. High levels of IFN-γ in fetal tissues can affect brain development and synapse formation, which can result in neurodevelopmental disorders [53]. Studies have shown that prenatal IFN-γ imbalances can be linked to autism [54]. This could help to prove the role of the immune abnormality with special emphasis on the impact of immunological variables connected to the maternal influence on autism development, comorbidities and severity [55]. The significant increase in CK and decrease in K+ in PPA and fluoxetine-treated rats demonstrate its role in energy impairment as a known etiological mechanism in ASD [56].

These significant positive and negative correlations shown in Figure 2 can collectively prove the contribution of oxidative stress, neuroinflammation, altered neurochemistry, apoptosis, and impaired energy metabolism as etiological mechanisms related to brain neurotoxicity in ASD.

The results of multilevel regression shown in Table 2 demonstrate that neuroinflammation (IF-γ) serves as a predictor variable for 95% of the increase in MDA, a sign of oxidative stress. This could support the idea that both pathways had a part in the etiology of ASD. Furthermore, oxidative stress and poor energy metabolism (K+, catalase, GST) are connected to 92.2% of the CK rise as predictive variables. Serotonin, dopamine, MDA, and K+, markers of neurochemistry, oxidative stress, and energy metabolism, account for 98.4% of the significant rise in caspase-3 as a proapoptotic marker.

ROC analysis presented in Table 3 shows the AUC together with the cut-off values of all test markers of the PA and fluoxetine group’s parameters; the total AUC was determined as a measure of the performance of PA or prenatal fluoxetine treatment. It is clearly seen that almost all the test markers under study can be used as excellent markers of both groups with AUC values equal to 1.

## 5. Conclusions

Fluoxetine affects the mother’s brain similarly to the way PA affects the brain of a newborn through increased oxidative stress, neuroinflammation and neurochemical alterations in the brain. We reported significant increase in MDA, IFN-γ, and caspase-3 with the significant decrease in GSH, GST, Catalase, K+, and CK and all measured neurotransmitters in both PPA and fluoxetine groups when compared with control group. Therefore, because of its developmental neurotoxicity, fluoxetine may be one of the causes of autism.

## Figures and Tables

**Table 1:** Mean ± SD of all the measured parameters in brain homogenate of all treated groups compared with the control.

Parameters	Groups	N	Min.	Max.	Mean ± S.D.	Percent Change	*p* Value ^a^	*p* Value ^b^
MDA (µmoles/mL)	Control	10	0.31	0.35	0.330 ± 0.014	100.00		0.001
	PPA	10	0.45	0.47	0.460 ± 0.009	139.39	0.001	
	FLX	10	0.35	0.37	0.360 ± 0.009	109.09	0.001	
GSH (ug/mL)	Control	10	33.20	37.55	36.19 ± 1.66	100.00		0.001
	PPA	10	28.99	31.20	30.04 ± 0.83	83.00	0.001	
	FLX	10	15.60	17.60	16.45 ± 0.86	45.45	0.001	
Catalase (U/dl)	Control	10	5.25	7.50	6.23 ± 0.87	100.00		0.001
	PPA	10	4.20	5.30	4.67 ± 0.45	75.01	0.002	
	FLX	10	3.89	5.20	4.59 ± 0.56	73.73	0.001	
GST (U/mL)	Control	10	13.22	18.22	15.47 ± 1.71	100.00		0.001
	PPA	10	11.22	13.80	12.55 ± 0.97	81.11	0.002	
	FLX	10	7.10	9.60	8.56 ± 0.84	55.33	0.001	
K+ (mmol/L)	Control	10	5.10	6.33	5.60 ± 0.53	100.00		0.001
	PPA	10	4.31	5.22	4.82 ± 0.34	86.01	0.010	
	FLX	10	2.50	3.44	2.94 ± 0.35	52.43	0.001	
CK (IU/L)	Control	10	250.33	311.80	284.91 ± 22.10	100.00		0.001
	PPA	10	275.55	300.22	287.07 ± 10.38	100.76	0.954	
	FLX	10	320.77	340.89	330.94 ± 7.17	116.16	0.001	
IF γ (pg/100 mg)	Control	10	87.50	90.44	89.18 ± 1.15	100.00		0.001
	PPA	10	111.44	115.33	113.50 ± 1.42	127.27	0.001	
	FLX	10	90.40	99.55	93.10 ± 3.28	104.39	0.013	
Norepinephrine (ng/100 mg)	Control	10	4.55	5.50	5.05 ± 0.32	100.00		0.001
	PPA	10	3.09	3.88	3.46 ± 0.27	68.55	0.001	
	FLX	10	3.99	5.11	4.82 ± 0.42	95.45	0.416	
Serotonin (ng/100 mg)	Control	10	5.95	7.10	6.63 ± 0.39	100.00		0.001
	PPA	10	3.99	4.42	4.23 ± 0.14	63.87	0.001	
	FLX	10	4.50	5.02	4.77 ± 0.18	72.03	0.001	
Dopamine (ng/100 mg)	Control	10	20.97	22.50	21.88 ± 0.50	100.00		0.001
	PPA	10	14.59	16.00	15.08 ± 0.51	68.92	0.001	
	FLX	10	15.17	17.09	16.08 ± 0.76	73.48	0.001	
Caspase-3 (pg/100 mg)	Control	10	108.40	113.18	110.83 ± 1.76	100.00		0.001
	PPA	10	138.90	145.30	142.56 ± 2.40	128.62	0.001	
	FLX	10	132.99	135.15	134.03 ± 0.93	120.93	0.001	

^a^*p* value between each group and the control group. ^b^ *p* value between all groups.

**Table 2:** Multiple regression using the stepwise method for measured parameters as a dependent variable.

Dependent Variable	Predictor Variable	Coefficient	*p* Value	Adjusted R^2^	95% CI	
					Lower	Upper
MDA	IF γ	0.005	0.000	0.950	0.004	0.006
K+	GSH	0.134	0.000	0.887	0.110	0.159
	CK	−0.017	0.010		−0.029	−0.005
CK	K+	−16.297	0.000	0.922	−22.599	−9.996
	Catalase	10.859	0.000		6.435	15.283
	GST	−3.047	0.021		−5.552	−0.542
IF γ	MDA	187.386	0.000	0.950	165.202	209.569
Serotonin	Caspase-3	−0.076	0.000	0.950	−0.085	−0.067
Dopamine	Caspase-3	−0.192	0.000	0.958	−0.222	−0.163
	GST	0.202	0.005		0.072	0.331
Caspase-3	Serotonin	−5.793	0.002	0.984	−9.090	−2.495
	Dopamine	−0.800	0.209		−2.107	0.507
	MDA	82.120	0.000		44.826	119.413
	K+	−2.005	0.011		−3.478	−0.532

*p* values of ≤0.05 were considered significant.

**Table 3:** ROC curve of all parameters in all groups.

Parameters	Groups	AUC	Cut-off Value	Sensitivity %	Specificity %	*p* Value	95% CI
MDA (µmoles/mL)	PPA	1.000	0.400	100.0%	100.0%	0.004	1.000–1.000
	FLX	0.972	0.345	100.0%	83.3%	0.006	0.889–1.056
GSH (ug/mL)	PPA	1.000	32.200	100.0%	100.0%	0.004	1.000–1.000
	FLX	1.000	25.400	100.0%	100.0%	0.004	1.000–1.000
Catalase (U/dl)	PPA	0.972	5.370	100.0%	83.3%	0.006	0.889–1.056
	FLX	1.000	5.225	100.0%	100.0%	0.004	1.000–1.000
GST (U/mL)	PPA	0.958	14.100	100.0%	83.3%	0.008	0.851–1.065
	FLX	1.000	11.410	100.0%	100.0%	0.004	1.000–1.000
K+ (mmol/L)	PPA	0.917	5.050	83.3%	100.0%	0.016	0.742–1.091
	FLX	1.000	4.270	100.0%	100.0%	0.004	1.000–1.000
CK (IU/L)	PPA	0.528	288.980	66.7%	50.0%	0.873	0.168–0.888
	FLX	1.000	316.285	100.0%	100.0%	0.004	1.000–1.000
IF g (pg/100 mg)	PPA	1.000	100.940	100.0%	100.0%	0.004	1.000–1.000
	FLX	0.972	90.300	100.0%	83.3%	0.006	0.889–1.056
Norepinephrine (ng/100 mg)	PPA	1.000	4.215	100.0%	100.0%	0.004	1.000–1.000
	FLX	0.694	5.030	83.3%	66.7%	0.262	0.377–1.012
Serotonin (ng/100 mg)	PPA	1.000	5.185	100.0%	100.0%	0.004	1.000–1.000
	FLX	1.000	5.485	100.0%	100.0%	0.004	1.000–1.000
Dopamine (ng/100 mg)	PPA	1.000	18.485	100.0%	100.0%	0.004	1.000–1.000
	FLX	1.000	19.030	100.0%	100.0%	0.004	1.000–1.000
Caspase-3 (pg/100 mg)	PPA	1.000	126.040	100.0%	100.0%	0.004	1.000–1.000
	FLX	1.000	123.085	100.0%	100.0%	0.004	1.000–1.000

## Data Availability

The data presented in the current manuscript.
